# Case of Ulcer of the Stomach

**Published:** 1827-01

**Authors:** John Hunter


### ULCERATION OF THE STOMACH.

*Case of Ulcer of the Stomach.* By JOHN HUNTER, jun. M.R.C.S.

M— C—, a married female, ætatis twenty-five, was suddenly attacked, on the 16th August, 1826, with excruciating pain in the præcordia, referred more especially to the umbilicus, which part was so exquisitely tender that she could scarcely bear it to be touched. Her pulse was seventy-two, small and soft; tongue clean; respiration but little affected, unless drawn deep. She had eaten a hearty dinner with the family about an hour before, consisting of duck, fruit-pie, plums, &c., and had just gone up-stairs, apparently in her usual health, when this seizure occurred. She took Tinct. Opii gtt. xxx. and shortly after vomited, bringing up from the stomach above a quart of aliment, partly digested. She drank freely of warm water to encourage the vomiting, and repeated it several times until it returned clear and free from smell, and the nausea had entirely ceased. This, however, afforded no relief, and she still continued to writhe and groan in great agony.

Fomentations were applied over the abdomen; the opiates repeated at short intervals, until she had taken 120 drops of the tincture, and two grains of the solid opium; and twenty ounces of blood drawn from the arm (producing faintness).

By these means she obtained a slight remission, but nothing like a total cessation from pain. She afterwards took two grains more of opium; and a blister was prescribed for the pit of the stomach, but applied to the left hypochondrium, from her dread of its increasing the pain.

On the following morning, her pulse was still seventy-two, rather fuller, but very weak; tongue furred; thirst excessive; great restlessness; pain constant, and the same tenderness about the navel; great depression of spirits, and a strong conviction on her mind that she should die. She had dozed occasionally during the night, but awoke on the slightest motion, with an aggravation of pain.

Ol. Ricini ℥ ss. was ordered, and repeated every hour till three ounces had been taken, without affecting the bowels or producing the least nausea. A large enema was then administered, but quickly returned, with a slight admixture of feces; and subsequently a dose of Calomel and Opium was given, after which she had several dark-coloured liquid stools. Leeches were also applied to the belly, and the fomentations continued.

In the course of the morning, she sank into an alarming state of collapse: her face, previously florid, became quite blanched; her extremities cold; the whole surface of the body bedewed with clammy sweats, and the pulse at the wrist not perceptible; but no abatement of the pain. From this condition she grew gradually worse till midnight, when she expired, just thirty hours after the attack. She remained perfectly sensible till near the time of her dissolution.

This young woman had dark hair and eyes, and a very delicate complexion, (though latterly her face was always flushed.) She was rather of an irritable disposition; had been married five years, and her husband treated her so ill that she had parted from him several times; but this did not seem to prey upon her spirits, which were very buoyant, and she moved about actively. She had complained of pain at the navel since the birth of a child above four years back, and attributed it to some mismanagement in her lying-in. Her hand was often pressed to the part for relief, and she was in the habit also of lying upon her face in bed, very proper for a woman living apart from her husband. Her

Her appetite was generally good: she preferred rich and high-seasoned food; took a great deal of salt and vinegar, and occasionally spirits; was never subject to nausea or vomiting. She was very fat, but supposed herself to be dropsical, on which account she was under the care of a physician, who had treated her with Pil. Hydrargyri, Pil. Scillæ C., and saline aperient medicines.

*Appearances on inspection of the body.—Abdomen:* The parietes and larger omentum loaded with fat; above two quarts of an opake serous fluid, with gelatinous flakes floating in it, in the cavity of the peritoneum. Increased vascularity of the peritoneal lining of the parietes. Erythematous patches on the surface of the small intestines, but no adhesion, though much lymph thrown out about them. The intestines filled with air, but devoid of feces; no appearance of inflammation on their internal coat. The principal seat of the complaint was in the stomach: in the anterior surface of this viscus, near the lesser curvature, was an ulcerated opening, of an irregular figure, nearly circular, about two-thirds of an inch in diameter, with a smooth polished edge externally, formed by the peritoneal coat only. The internal margin of this aperture was composed of the mucous and muscular coats united; the upper half of its circumference thickened, white, and shining; the lower half irregular. No contiguous inflammation, either of the internal or external coats. The liver was clayey and exsanguinous. The gall-bladder filled with bile. The spleen somewhat enlarged. The pancreas and kidneys healthy.

*Thorax:* The lungs presented their usual appearance. A layer of fat on the anterior surface of the heart quite concealed its muscular structure; and in the anterior mediastinum was a further deposit of it.

The ulcer of the stomach exactly corresponds with another specimen of the same disease in the museum of St. Thomas's Hospital, where also this stomach is deposited. It is probable, from the long existence of pain at the navel, that its action was very gradual; and, from the superabundance of fat throughout every part of the body, it appears to have formed no impediment to the process of digestion. The opening of this ulcer through the peritoneal coat occasioned, without doubt, the violent symptoms preceding death.

*Mincing-lane; November* 1826.

